# Diagnostic tools for alzheimer’s disease dementia and other dementias: an overview of diagnostic test accuracy (DTA) systematic reviews

**DOI:** 10.1186/s12883-014-0183-2

**Published:** 2014-09-24

**Authors:** Ingrid Arevalo-Rodriguez, Omar Segura, Ivan Solà, Xavier Bonfill, Erick Sanchez, Pablo Alonso-Coello

**Affiliations:** Division of Research, Fundación Universitaria de Ciencias de la Salud, Hospital de San José/Hospital Infantil de San José, Bogotá, DC Colombia; Pediatrics, Obstetrics & Gynecology and Preventive Medicine Department, Universitat Autonoma de Barcelona, Barcelona, Spain; Iberomerican Cochrane Centre, Clinical Epidemiology and Public Health Department, Institute of Biomedical Research (IIB Sant Pau), Spain, CIBER de Epidemiología y Salud Pública (CIBERESP), Barcelona, Spain; Iberoamerican Cochrane Centre - Institute of Biomedical Research (IIB Sant Pau), CIBER Epidemiología y Salud Pública (CIBERESP), Spain - Universitat Autònoma de Barcelona, Barcelona, Spain; Department of Neurosciences and Memory Clinic Unit, Fundacion Universitaria de Ciencias de la Salud, Bogotá, Colombia

**Keywords:** Diagnosis, Dementia, Alzheimer’s disease dementia, Systematic review, PRISMA checklist, AMSTAR tool

## Abstract

**Background:**

Dementia includes a group of neurodegenerative disorders characterized by progressive loss of cognitive function and a decrease in the ability to perform activities of daily living. Systematic reviews of diagnostic test accuracy (DTA) focus on how well the index test detects patients with the disease in terms of figures such as sensitivity and specificity. Although DTA reviews about dementia are essential, at present there is no information about their quantity and quality.

**Methods:**

We searched for DTA reviews in MEDLINE (1966–2013), EMBASE (1980–2013), *The Cochrane Library* (from its inception until December 2013) and the Database of Abstracts of Reviews of Effects (DARE). Two reviewers independently assessed the methodological quality of the reviews using the AMSTAR measurement tool, and the quality of the reporting using the PRISMA checklist. We describe the main characteristics of these reviews, including basic characteristics, type of dementia, and diagnostic test evaluated, and we summarize the AMSTAR and PRISMA scores.

**Results:**

We selected 24 DTA systematic reviews. Only 10 reviews (41.6%), assessed the bias of included studies and few (33%) used this information to report the review results or to develop their conclusions Only one review (4%) reported all methodological items suggested by the PRISMA tool. Assessing methodology quality by means of the AMSTAR tool, we found that six DTA reviews (25%) pooled primary data with the aid of methods that are used for intervention reviews, such as Mantel-Haenszel and separate random-effects models (25%), while five reviews (20.8%) assessed publication bias by means of funnel plots and/or Egger’s Test.

**Conclusions:**

Our assessment of these DTA reviews reveals that their quality, both in terms of methodology and reporting, is far from optimal. Assessing the quality of diagnostic evidence is fundamental to determining the validity of the operating characteristics of the index test and its usefulness in specific settings. The development of high quality DTA systematic reviews about dementia continues to be a challenge.

**Electronic supplementary material:**

The online version of this article (doi:10.1186/s12883-014-0183-2) contains supplementary material, which is available to authorized users.

## Background

Population ageing is generating a considerable increase in chronic and neurodegenerative diseases, as well as severe consequences for global public health [1,2]. Dementia includes a group of neurodegenerative disorders characterized by progressive loss of cognitive function as well as the ability to perform activities of daily living, sometimes accompanied by neuropsychiatric symptoms [3]. Criteria for dementia diagnosis include a deficit in one or more cognitive domains that is severe enough to impair functional activities, and is progressive over a period of at least six months and not attributable to any other brain disease [4,5]. The presence of cognitive impairment, a fundamental part of the dementia profile, could be detected through a combination of history, clinical examination, and objective cognitive assessment such as a brief mental assessment or comprehensive neuropsychological testing [6,7]. At present, there is a trend towards incorporating biomarker tests into dementia diagnosis criteria, such as amyloid-β protein accumulation, neuronal injury, synaptic dysfunction, and neuronal degeneration [8-10].

Systematic reviews of diagnostic test accuracy (DTA) focus on how well an index test detects patients with the disease in terms of figures such as sensitivity and specificity. DTA reviews present summarized information to consumers (such as clinicians, stakeholders, guideline developers and patients) about which test should be used over another as the initial step in a diagnostic pathway or as an add-on element to confirm the presence of the target disease. Although the methodology for performing DTA reviews is constantly evolving, organizations such as the Cochrane Collaboration have published methodological guidance with basic requirements to develop these kinds of reviews [11].

Recently, we evaluated the quality of clinical practice guidelines for diagnosing dementia and found a wide variety in terms of quality of evidence as well as the strength of the recommendations provided [12]. Although DTA reviews are an essential part of any clinical guidelines, at present there is no information about the quantity and quality of dementia DTA reviews. This information could help clinicians and stakeholders provide adequate management and appropriate care for these patients, in line with the rise in dementia and its expected burden on health systems.

The objective of this study was to evaluate the quality (in terms of rigor in conduct and reporting) of DTA systematic reviews related to diagnostic tools for Alzheimer’s disease dementia (ADD) and other dementias. These tools included brief cognitive tests, biomarkers, and neuropsychological assessment, and they were assessed by means of standardized tools, as well as by describing the tests evaluated and their main characteristics.

## Methods

We produced a protocol for the review (available from the authors on request) detailing the proposed review methods. We searched in MEDLINE (1966–2013), EMBASE (1980–2013), *The Cochrane Library* (from its inception until December 2013) and the Database of Abstracts of Reviews of Effects (DARE), by means of a predesigned search strategy adapted to each database (Additional file 1), in order to identify diagnostic systematic reviews focused on the test accuracy of diagnostic tools for dementia, ADD or other dementias (e.g. vascular dementia, frontotemporal dementia, Lewy bodies, and Parkinson dementia). We checked the reference lists of the selected studies for additional references, and excluded congress abstracts and references with insufficient information.

Two reviewers independently assessed the eligibility of the results and extracted data from the selected studies. In this overview we included systematic reviews of diagnostic studies that focused on the accuracy of tests for dementia. Only reviews that used a systematic approach, included adult patients aged over 50 suspected of having dementia, and estimated the accuracy of the assessed test (i.e. providing sensitivity and specificity figures) were considered. We used a predefined data extraction form to extract descriptive information including year of publication, type of studies included, and clinical reference standard, and whether a checklist was used to evaluate the methodological quality of primary studies (such as the Quality Assessment of Diagnostic Accuracy Studies, QUADAS [13,14]).

After this was done, two reviewers independently assessed the methodological quality of the selected reviews using the Assessment of Multiple Systematic Reviews (AMSTAR) measurement tool [15], tailored to the characteristics of DTA systematic reviews (Additional file 1). They also assessed the quality of the reporting using the Preferred Reporting Items for Systematic reviews and Meta-Analyses (PRISMA) checklist [16]. We resolved disagreements through discussion. In this article we describe the main characteristics of the selected reviews, including basic characteristics (e.g. reference standard used and diagnostic bias reported). We also describe the type of dementia and index test evaluated, as well as AMSTAR and PRISMA scores by item.

## Results

**Figure 1:**
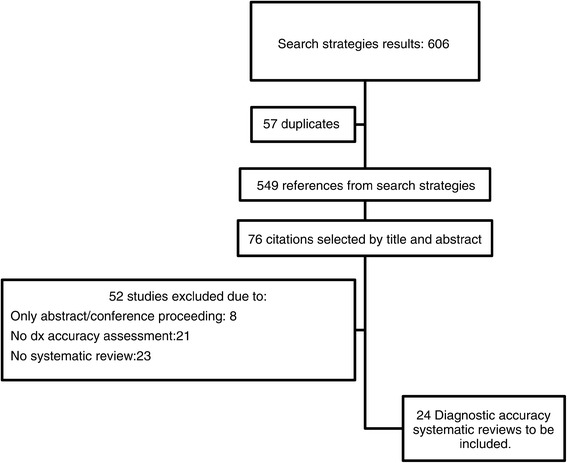
**Flowchart of the systematic search.**

We retrieved a total of 549 citations after excluding duplicates and initially selected a total of 76 references for full review. We excluded 52 articles because they did not provide diagnostic accuracy information, presented a narrative overview about dementia, or did not have enough information to evaluate their quality (i.e. congress abstracts) (Additional file 1). Finally, we selected 24 DTA systematic reviews [17-40], with a median sample size of 2,190 patients (range from 160 to 26,019) (Figure 1 and Additional file 1).

Ten reviews (41.6%) focused on mild cognitive impairment (MCI), an early stage of dementia, either for detection or conversion to full dementia, while nine reviews (37.5%) focused on ADD, and eight on dementia in general. Eight out of 24 DTA reviews (33%) included more than one subtype of dementia. Seven studies (29%) had less than 1,000 patients, and a similar number did not report the total number of patients derived from primary studies, while nine (37.5%) of the reviews included less than 10 studies (Additional file 1).

The reviews selected included mostly cross-sectional and cohort studies, with a median of 19.5 primary studies included (range from two to 233 primary studies). The index tests most frequently evaluated were cognitive tests (nine DTA reviews), followed by PET/SPECT and serum levels of Total Tau and P-Tau (six DTA reviews each). Several reference standards were used to validate dementia diagnoses, with NINCS-ARDRA and DSM-IV being the most common (11 and nine reviews, respectively). Four DTA reviews did not indicate the reference standard used to evaluate the validity of dementia diagnoses. Table 1 shows the selected reviews by type of dementia and diagnostic tool evaluated.

**Table 1:** **DTA systematic reviews about dementia by type of dementia and diagnostic tool evaluated**

	**MMSE**	**Other cognitive tests**	**PET/ SPECT**	**CSF Aβ**_**42**_	**P –Tau/T-Tau**	**FDG/PIB uptake on PET**	**MRI/ CT**	**Other diagnostic tools**
**ADD**			Bloudek [19]	Bloudek [19]	Bloudek [19]	Bloudek [19]	Bloudek [19]	
			Dougall [22]		Mitchell [28]	Matchar [27]		
			Ferrante [24]			Patwardhan [34]		
**DLB**			Yeo [40]		Van Harten [36]			Papathanasiou [33]
								Treglia [35]
								Yeo [40]
**VaD**			Dougall [22]		Van Harten [36]		Beynon [18]	Yeo [40]
			Yeo [40]					
**FTD**			Dougall [22]		Van Harten [36]			Yeo [40]
			Yeo [40]					
**Dementia in general**	Mitchell [29]	Appels [17]	Ferrante [24]			Matchar [27]		
		Carnero [20]						
		Crawford [21]						
		Mitchell [30]						
		Mitchell [31]						
**MCI**	Lischka [25]	Ehreke [23]	Yuan [38]	Monge [32]	Mitchell [28]	Zhang [39]	Yuan [38]	
	Lonie [26]	Lischka [25]		van Rossum [37]	Monge [32]			
	Mitchell [29]	Lonie [26]			van Rossum [37]			

*Abbreviations:* *Aβ*_*42*_ 42 aminoacid form of amyloid-β, *ADD* Alzheimer’s Disease Dementia, *CT* Computed tomography, *DLB* Dementia with Lewy Bodies, *FDG-PET* PET using 2-Fluro-deoxy D-glucose, *FTD* Fronto-Temporal Dementia, *MCI* Mild cognitive Impairment, *MMSE* Mini-Mental State Examination, *MRI* Magnetic Resonance Imaging, *PIB-PET* 11 C-Pittsburgh Compound B- positron emission tomography, *Ptau* Phosphorylated Tau, *SPECT* Single photon emission computed tomography, *Ttau* Total Tau, *VaD* Vascular Dementia.

Only 10 reviews (41.6%) assessed the methodological quality of primary studies, with QUADAS-I being the most commonly used tool for assessing risk of bias of primary studies (six studies, 60%). Patient spectrum and incorporation bias were the most frequent biases reported by review authors. Four reviews assessed the methodological quality of primary studies by means of the STARD tool (16%), which is intended to assess reporting quality. None of the DTA reviews reported results related to inconclusive results, adverse events, or the use of resources related to index tests in an explicit way. Eleven reviews (45.8%) reported the sources of funding or support to perform the DTA review with most of them being government sources.

**Figure 2:**
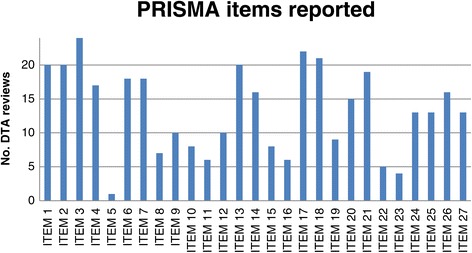
**PRISMA items reported by DTA reviews about dementia.** Notes: Item 1 = Identify the report as a systematic review; Item 2 = Provide a structured summary; Item 3 = Describe the rationale of the review; Item 4 = Provide an explicit statement of questions being addressed; Item 5 = Indicate if a review protocol exists; Item 6 = Specify study characteristics; Item 7 = Describe all information sources in the search and date last searched; Item 8 = Present full electronic search strategy; Item 9 = State the process for selecting studies; Item 10 = Describe method of data extraction; Item 11 = List and define all variables for which data were sought; Item 12 = Describe methods used for assessing risk of bias of individual studies; Item 13 = State the principal summary measures; Item 14 = Describe the methods of handling data and combining results; Item 15 = Specify any assessment of risk of bias that may affect the evidence; Item 16 = Describe methods of additional analyses; Item 17 = Give numbers of studies screened, assessed for eligibility, and included in the review; Item 18 = For each study, present characteristics for which data were extracted; Item 19 = Present data on risk of bias of each study; Item 20 = For all outcomes present simple summary data, effect estimates and confidence intervals; Item 21 = Present the main results of the review; Item 22 = Present results of any assessment of risk of bias across studies; Item 23 = Provide results of additional analyses; Item 24 = Summarize the main findings; Item 25 = Discuss limitations at study and outcome level and at review-level; Item 26 = Provide a general interpretation of the results and implications for future research; Item 27 = Describe sources of funding for the systematic review.

With regards to the PRISMA checklist, all selected reviews (100%) described the rationale for the review (Item 3), and 20 (83.3%) identified themselves as systematic reviews (Item 1). Twenty-two reviews (91.6%) reported the number of studies screened, assessed for eligibility, and included in the review, by means of a flow chart (Item 17) and 21 (87.5%) presented characteristics of studies and provided citations (Item 18). However, only one study (4.1%) reported a review protocol (Item 5), four (16.6%) reported results of additional analysis (Item 23), and five (20.8%) presented results of risk of bias assessment across studies as publication bias or selective reporting within studies (Item 22). Only one review reported all methodological items suggested by the PRISMA tool (Items 5 to 16) (Figure 2).

**Figure 3:**
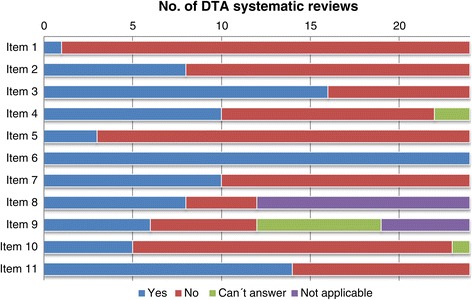
**Results of AMSTAR assessment- DTA systematic reviews about dementia.** Notes: Item 1 = Priori design; Item 2 = Duplicate study selection/data extraction; Item 3 = Comprehensive literature search; Item 4 = inclusion criterion -status of publication; Item 5 = list of studies provided; Item 6 = characteristics of the included studies provided; Item 7 = scientific quality of the included studies assessed & documented; Item 8 = scientific quality of the included studies in formulating conclusions; Item 9 = methods used to combine the findings of studies appropriate; Item 10 = publication bias assessed; Item 11 = conflict of interest stated.

With respect to the quality of conduct in terms of the AMSTAR tool, 21 reviews (87.5%) did not provide a list of included/excluded studies and 16 (66.6%) did not report duplicate study selection/data extraction (Figure 3). All reviews reported the characteristics of included studies (100%). Six DTA reviews (25%) pooled primary data by means of methods that are used for intervention reviews such as Mantel-Haenszel and separate random-effects models, while in seven reviews (29%) it was not possible to determine which methods were used to combine the numerical results. Five reviews (20.8%) assessed publication bias by means of funnel plots and/or Egger’s Test. Fourteen of these DTA reviews (58.3%) reported possible conflicts of interest. As mentioned above, only 10 reviews (41.6%) assessed the bias of included studies, and only eight (33%) used this information to report the review results or reach their conclusions.

## Discussion

Our review of DTA systematic reviews about dementia shows several areas for improvement. First, we had to exclude a significant number of reviews focused not on the accuracy of the test (i.e. sensitivity and specificity figures), but instead presenting information about the average differences between case and control groups. In these reviews, the authors gathered information about Phase I diagnostic studies, evaluating the differences (for example, in terms of difference of means) between a group of subjects with the disease and healthy controls [41]. These studies are essential for an adequate and full assessment of any diagnostic tool, but cannot show if the test distinguishes between those with and without the target condition. Authors of future reviews should be careful in appraising these studies due to the higher risk of bias (for instance, the wide use of cases and controls design) and their limitations in decision-making processes.

In relation to the basic characteristics of dementia DTA systematic reviews, we noticed that a significant number of reviews were focused on mild cognitive impairment (MCI). Identification of early forms of dementia has become an important topic because some interventions have been claimed to be effective in slowing or stopping the cognitive decline when they are administered in earlier stages of dementia, but these findings are still being investigated [42-45]. Similarly, it is interesting that a significant number of reviews focused on cognitive tests, which are the first line of detection for cognitive impairment in dementia. At present it is unclear which cognitive test should be the instrument of choice for initial dementia screening in population-based, primary and secondary settings, due to rising criticism of the role of traditional tests such as Mini-Mental State examination (MMSE) [12].

Our assessment of these DTA reviews reveals that their quality, in terms of both methodology and reporting, is far from optimal. We found that more than half of the included reviews did not provide a quality assessment of the primary studies, and therefore information of an unknown quality was gathered and even numerical pooled results were provided. Assessing the quality of diagnostic evidence is fundamental to determining the validity of the operating characteristics of the index test, and its usefulness in specific settings [46]. Four reviews (16%) did not report the reference standards they used to evaluate the accuracy of the different tests appraised, while others reported STARD scores as an evaluation of methodological quality. Only 13 of the 24 reviews (54%) described the limitations of the information gathered, and in only eight cases (33%) was the quality of this information considered in the conclusions. Likewise, AMSTAR and PRISMA items in conjunction showed an almost complete absence of a priori protocols presenting pre-specified methodological plans. The importance of pre-specified protocols has been established in intervention reviews as well as in clinical trials of pharmacological interventions. Diagnostic tests for dementia, such as FDG-PET and Tau-AB_42_, can be understood as medical technologies that can be affected by conflicts of interest. The availability of protocols at the beginning of any study not only ensures rigor in development, but also avoids conducting unnecessary research [47].

In our study we also identified drawbacks in developing DTA systematic reviews related to the application of statistical methods generally used in intervention systematic reviews. For example, when the methods used for pooling numerical information were assessed, we identified three reviews that used Der Simonian-Laird random effects models, instead of methods highly recommended in these cases such as bivariate models [48]. Some authors have asserted that the use of inadequate statistical techniques to deal with diagnostic information could lead to failures in managing the combined results of sensitivity and specificity [48-51]. Similarly, some reviews used the I^2^ statistic to illustrate the heterogeneity between the analyzed studies. Heterogeneity is a common issue in accuracy reviews, due to factors such as threshold used, prevalence of the target condition in the sample selected, and settings of test evaluation [52], but at present, there are no defined standards for how diagnostic heterogeneity should be measured and managed in DTA reviews [53].

In the same way, we identified that evaluation of publication bias remains a problem in dementia DTA reviews. In our study, 18 reviews (75%) did not provide information about this bias, but it is unclear if the authors simply omitted this evaluation or if they decided not to assess this topic due to lack of suitable analysis methods. Three additional cases (12.5%) used funnel-plot figures or statistical tests (for instance, Egger’s test). While these methods are highly useful in intervention systematic reviews, several research studies have shown that their use in the field of DTA reviews, usually by means of diagnostic odds ratios (DOR), can generate misleading results [54].

Our study has some limitations. One of these is related to the tools used to evaluate systematic reviews (such as PRISMA and AMSTAR), focused mainly on intervention reviews. In order to correctly use the AMSTAR tool we developed tailored definitions to adequately reflect the most accepted methodology of DTA systematic reviews. However, it is important to encourage discussion about how current tools and methodologies (for example, overview methodology) can be applied or adapted to developing DTA studies. A second difficulty that we found was the large number of diagnostic reviews reported only in abstract form, which had to be excluded because of the absence of information to allow for a full assessment of their elements. We believe that these “ongoing” studies reflect the growing interest in the diagnosis of dementia, as well as the need for comprehensive discussion about dementia diagnosis tools. Finally, our search strategy was specific and did not include terms related to MCI. Our findings related to this early stage of dementia might be incidental and not reflect all possible DTA reviews in this area.

## Conclusions

Development of systematic reviews of diagnostic test accuracy for dementia remains a difficult task. However, an increasing number of health professionals require information about the quality of diagnostic technologies due to their role in detecting, staging and monitoring. We believe that some recent initiatives might help improve methodology and reporting quality in DTA reviews on dementia [11,48,55]. In the near future, high quality DTA reviews could play an important role in helping clinicians, policy-makers and even patients to make informed decisions for the diagnosis of this prevalent disease.

## Additional file

Additional file 1: This file includes the following information: 1. Search strategy used on MEDLINE. 2. Operational definitions of AMSTAR’s Items for DTA reviews about dementia. 3. DTA systematic reviews about dementia- Descriptive information. 4. DTA systematic reviews about dementia- AMSTAR items. 5. DTA systematic reviews about dementia- PRISMA items. 6. List of excluded studies.

## Abbreviations

7MS: Seven-minute screen
99mTc-HMPAO 99 m: Technetium-hexamethyl-propylenamine oxime
Aβ _1–42_: 42 aminoacid form of amyloid-β
ACE: Addenbrooke’s cognitive examination
ADAS-CoG: Alzheimer’s disease assessment scale- cognitive
ADD: Alzheimer’s disease dementia
ADDTC: State of california AD diagnostic and treatment centre criteria
BKSCA-R: Brief Kingston standardized cognitive assessment- revised
BVRT: Benton’s visual retention test
CAMCI: Chinese abbreviated mild cognitive impairment test
CAMCOG: Cambridge cognitive examination
CASI: Cognitive abilities screening instrument
CAST: Cognitive assessment screening test
CCCE: Cross-cultural cognitive examination
CCSE: Cognitive capacity screening examination
CERAD: Consortium to Establish a registry for Alzheimer’s Disease
CSF: Cerebrospinal fluid
CSI-D: Community screening instrument for dementia
CT: Computed tomography
DaTSCAN: DaT, 123 I-FP-CT
DLB: Dementia with Lewy Bodies
DSM: Diagnostic and statistical manual of mental disorders
DTA: Diagnostic test accuracy
FDG-PET: PET using 2-Fluro-deoxy D-glucose
FTD: Fronto-temporal dementia
ICD-10: International statistical classification of diseases and related health problems, 10th revision
IQCODE: Informant questionnarie on cognitive decline in the elderly
IST: Isaacs set test
M@T: Memory alteration test
MCI: Mild cognitive impairment
MDRS: Mattis dementia rating scale
MFI: Mental function index
MIBG: 123 I-metaiodobenzylguanidine scintigraphy
MIS: Memory impairment screen
MMSE: Mini-mental state examination
MOCA: Montreal cognitive assessment
MRI: Magnetic resonance imaging
NCSE: Neurobehavioral cognitive screening examination
NINCDS-ADRDA: National Institute of Neurological and Communicative Disorders and Stroke and the Alzheimer’s Disease and Related Disorders Association
NINDS-AIREN: National Institute of Neurological Disorders and Stroke and Association Internationale pour la Recherche et l’Ensignement en Neurosciences
NUCOG: Neuropsychiatry unit cognitive assessment tool
PIB-PET: 11 C-Pittsburgh Compound B- positron emission tomography
Ptau: Phosphorylated tau
RBANS: Repeatable battery for the assessment of neuropsychological status
RUDAS: Rowland universal dementia assessment scale
SPECT: Single photon emission computed tomography
STMS: Short test of mental status
TMT B: Trail making Test Part B
Ttau: Total tau
VaD: Vascular dementia
