# Supplementary material for: Diagnostic tools for alzheimer’s disease dementia and other dementias: an overview of diagnostic test accuracy (DTA) systematic reviews
Source: BMC Neurol. 2014 Sep 24;14:183. doi: 10.1186/s12883-014-0183-2 (PMC4189736; doi:10.1186/s12883-014-0183-2)
Supplement: Additional file 1: — This file includes the following information: 1. Search strategy used on MEDLINE. 2. Operational definitions of AMSTAR’s Items for DTA reviews about dementia. 3. DTA systematic reviews about dementia- Descriptive information. 4. DTA systematic reviews about dementia- AMSTAR items. 5. DTA systematic reviews about dementia- PRISMA items. 6. List of excluded studies. [file 12883_2014_183_MOESM1_ESM.docx]

**Additional file 1**

**1. Search strategy used on MEDLINE**

1. "Dementia"[Mesh] OR dement*[ti] OR senil*[ti] OR Alzheimer*[ti] OR AD [tiab]
2. sensitiv* [Title/Abstract] OR sensitivity and specificity [MeSH Terms] OR diagnos* [Title/Abstract] OR diagnosis [MeSH:noexp] OR diagnostic * [MeSH:noexp] OR diagnosis,differential [MeSH:noexp] OR diagnosis [Subheading:noexp]
3. 1 AND 2
4. "Meta-Analysis as Topic"[Mesh] OR "Meta-Analysis"[Publication Type] OR systematic review [tiab] OR meta analysis[tiab]
5. 4 AND 3

**2. Operational definitions of AMSTAR´s Items for DTA reviews about dementia.**

The authors followed the Cochrane Handbook for DTA reviews in order to provide operational definitions of AMSTAR´s items adequate for diagnostic reviews on dementia.

| **AMSTAR ITEM** | **Operational definition** |
| --- | --- |
| 1. Was an ’a priori’ design provided? | The research question and inclusion criteria should be established before the conduct of the review. |
| 1. Was there duplicate study selection and data extraction? | There should be at least two independent data extractors and a consensus procedure for disagreements should be in place. |
| 1. Was a comprehensive literature search performed? | At least two electronic sources should be searched. The report must include years and databases used (e.g. Central, EMBASE, and MEDLINE). Key words and/or MESH terms must be stated and where feasible the search strategy should be provided. All searches should be supplemented by consulting current contents, reviews, textbooks, specialized registers, or experts in the particular field of study, and by reviewing the references in the studies found. Note: Include MEDLINE, EMBASE, DARE, MEDION, search for grey literature and search on reference list of primary studies. |
| 1. Was the status of publication (i.e. grey literature) used as an inclusion criterion? | The authors should state that they searched for reports regardless of their publication type. The authors should state whether or not they excluded any reports (from the systematic review), based on their publication status, language etc. |
| 1. Was a list of studies (included and excluded) provided? | A list of included and excluded studies should be provided. |
| 1. Were the characteristics of the included studies provided? | In an aggregated form such as a table, data from the original studies should be provided on the participants, interventions and outcomes. The ranges of characteristics in all the studies analyzed e.g. age, race, sex, relevant socioeconomic data, disease status, duration, severity, or other diseases should be reported.  Note: Type of studies (cross-sectional, CyC, nested CyC, cohorts, ECC´s control group).  Spectrum of patients, index test (Add-on, Replacement), prevalence estimated/range, target condition defined, clinical reference standard. |
| 1. Was the scientific quality of the included studies assessed and documented? | 'A priori' methods of assessment should be provided (e.g., for effectiveness studies if the author(s) chose to include only randomized, double-blind, placebo controlled studies, or allocation concealment as inclusion criteria); for other types of studies alternative items will be relevant.  Note: QUADAS I-II, but other checklist could be accepted. STARD and similar doesn’t evaluate risk of bias (quality of report). Operational definitions of QUADAS items are desirables. |
| 1. Was the scientific quality of the included studies used appropriately in formulating conclusions? | The results of the methodological rigor and scientific quality should be considered in the analysis and the conclusions of the review, and explicitly stated in formulating recommendations.  Note: Include sensitivity analysis or subgroup analysis. |
| 1. Were the methods used to combine the findings of studies appropriate? | Heterogeneity is common on DTA reviews. Consider for meta-analysis a random effect model. Adequate models recommended by Cochrane Collaboration include Bivariate model and Hierarchical SROC.  **I-square is not recommended in DTA reviews.**  Sources of heterogeneity on dementia studies:  1. Index test  a. Thresholds  b. Technical features (including different versions of the test)  c. Operator characteristics e.g. training  2. Target disorder  a. Reference standard/s used: DSM definition, ICD definition, NINDS-ARDRA, or other classification, including pathological definitions; and operationalisation of these classifications (e.g. individual clinician / algorithm / consensus group).  b. Spectrum of target disorder (may also depend on study design)  3. Target population  a. Sociodemographic characteristics  b. Other characteristics e.g ApoE status, definition and duration of MCI at baseline (if applicable)  c. Prevalence in different settings  d. Treatment, previous or current interventions  4. Study quality  a. Types of studies: see below  b. Blinding. Prior clinical information will increase accuracy of the index test.  c. Time from index test to reference standard (measured in days or weeks for cross-sectional studies)  d. Duration of follow-up (measured in years for delayed-verification studies)  e. Loss to follow-up |
| 1. Was the likelihood of publication bias assessed? | Quantitative methods for exploring reporting bias are not well established for studies of DTA. Specifically, funnel plots of the DOR versus the standard error of this estimate will not be considered. |
| 1. Was the conflict of interest stated? | Potential sources of support should be clearly acknowledged in both the systematic review and the included studies. |

**3. DTA systematic reviews about dementia- Descriptive information.**

| **Review** | **Type of dementia** | **Index test** | **Reference standard** | **Years included in search strategy** | **Number of primary studies included** | **Total number of patients** |
| --- | --- | --- | --- | --- | --- | --- |
| Appels 2010 [[15](#_ENREF_15)] | Dementia | ACE, ADAS-Cog, CAMCOG, MDRS, MOCA, NCSE, RBANS, 7MS | Various | 1966-2009 | 17 | 2394 |
| Beynon 2012 [[16](#_ENREF_16)] | VaD + Mixed dementia vs. ADD | CT, MRI | Autopsy, NINCDS-ADRDA, NINDS-AIREN, DSM III, DSM III-R, ICD-10, ADDTC, Mixed dementia criteria, Hachinsky ischemic score. | Inception- February 2011 | 38 | 4377 |
| Bloudek 2011 [[17](#_ENREF_17)] | ADD | MRI, CT, SPECT, FDG-PET, CSF P-Tau, CSF Aβ_42_ | NINCDS-ADRDA, autopsy | January 1990- March 2010 | 119 | Not reported |
| Carnero 2003 [[18](#_ENREF_18)] | Dementia | FDG-PET | Not reported | January 2000- March 2003 | 2 | 160 |
| Crawford 2012 [[19](#_ENREF_19)] | Dementia | ACE, ACE-R | NINCDS-ADRDA, DSM-IV, NINDS-AIREN, Mattis dementia rating scale, FTD Consensus | 2000- April 2010 | 9 | 1550 |
| Dougall 2004 [[20](#_ENREF_20)] | ADD+ VaD + FTD | 99m Tc- HMPAO-SPECT. | NINCDS-ADRDA, DSM-III-R/IV, Hachinski scale | January 1985- December 2002 | 60 | Unclear |
| Ehreke 2010 [[21](#_ENREF_21)] | MCI | Clock Drawing Test | Not provided | Inception-July 2008 | 9 | 1634 |
| Ferrante 2004 [[22](#_ENREF_22)] | ADD + Dementia | SPECT | Clinical reference, Histopathology | No reported | 32 | Not reported |
| Lischka 2012 [[23](#_ENREF_23)] | MCI | Screening tools (ADAS-COG, BVRT, CAMCOG, CCSE, IST, S-MMSE, ACE, ACE-R, CAMCI, IQCODE, among others) | Clinical examination at follow-up | 1995-2011 | 12 | Not reported |
| Lonie 2009 [[24](#_ENREF_24)] | MCI | Cognitive screening measures ( ACE-R, MOCA, CERAD, CAMCOG, MMSE, CDT, M@T, SIS, DemTec, ABCS, STMS, among others) | Not provided | 1999-2008 | 30 | Unclear |
| Matchar 2001 [[25](#_ENREF_25)] | Dementia + MCI + ADD | FDG-PET | Histopathology Clinical diagnosis | 1995-2001 | 15 | 975 |
| Mitchell 2009a [[27](#_ENREF_27)] | Dementia + MCI | MMSE | DSM (III, III-R, IV), NINCDS-ADRDA, AGECAT, CAMDEX, Expert Dx, Mayo Clinic Criteria (for MCI) | Inception-2008 | Dementia specialized settings=13  Dementia non-specialized settings= 21  MCI=5 | Dementia specialized settings=5369  Dementia non-specialized settings= 26019  MCI=1587 |
| Mitchell 2009b [[26](#_ENREF_26)] | ADD + MCI | P-Tau | NINCDS-ADRDA Mayo clinic Criteria | Inception -2009 | 51 | 4597 |
| Mitchell 2010a [[28](#_ENREF_28)] | Dementia | Multi-domain screening methods | DSM (III, III-R, IV), NINCDS-ADRDA, AGECAT, CAMDEX, ICD-10 | Inception -July 2009 | 44 | Community= 5022  Primary care=4440  Secondary care=9959 |
| Mitchell 2010b [[29](#_ENREF_29)] | Dementia | Single-domain screening methods | DSM (III, III-R, IV), NINCDS-ADRDA, AGECAT, CAMDEX, ICD-10 | Inception -July 2009 | 20 | Community= 3572  Primary care=4875  Secondary care=7773 |
| Monge 2010 [[30](#_ENREF_30)] | MCI | CSF Aβ_42_, T-Tau, P-Tau. | DSM-IV, NINDS-ADRDA | 1999-2008 | 12 | 818 |
| Papathanasiou 2012 [[31](#_ENREF_31)] | DLB | DaTSCAN | Clinical Dx, Histopathology post-mortem | Inception- August 2011 | 4 | 419 |
| Patwardhan 2004 [[32](#_ENREF_32)] | ADD | FDG-PET | NINCDS-ADRDA, DSM, histopathology | 1989-2003 | 15 | Not provided |
| Treglia 2012 [[33](#_ENREF_33)] | DLB | MIBG scintigraphy | Clinical criteria | Inception-May 2010 | 8 | 346 |
| Van Harten 2011 [[34](#_ENREF_34)] | DLB + FTD + VaD vs ADD | T-Tau, P-Tau. | Various | Inception- July 2010 | P-tau: DLB= 9; FTD=10; VaD=8.  For T-tau : DLB= 19; FTLD=22; VaD=23 | For P-tau: DLB= 741; FTLD=783; VaD=498  For T-tau: DLB= 681; FTLD=1794; VaD=1815 |
| van Rossum 2010 [[35](#_ENREF_35)] | MCI | CSF Aβ_42_, T-Tau, P-Tau. | Not provided | No provided | 19 | 1986 |
| Yeo 2013 [[38](#_ENREF_38)] | DLB + FTLD + VaD vs ADD | Tc-HMPAO, SPECT, TcECD SPECT | Pathological, McKeith criteria | January 1985- May 2012 | 49 | Not reported |
| Yuan 2009 [[36](#_ENREF_36)] | MCI | FDG-PET, SPECT, MRI | NINCDS-ADRDA, DSM-IV, Histopathology | January 1990-April 2008 | 6 FDG-PET  8 SPECT  10 MRI | 1112 |
| Zhang 2012 [[37](#_ENREF_37)] | MCI | FDG-PET, PIB-PET | Histopathology, NINCDS-ADRDA, DSM-IV, Petersen criteria | January 2001- July 2001 | 7 FDG-PET  6 PIB-PET | 492 |

*Abbreviations: 7MS= Seven-Minute Screen; 99mTc-HMPAO= 99m Technetium-hexamethyl-propylenamine oxime; Aβ _1-42_=42 aminoacid form of amyloid-β; ACE= Addenbrooke´s Cognitive Examination; ADAS-CoG= Alzheimer´s Disease Assessment Scale- Cognitive; ADD= Alzheimer´s Disease Dementia; ADDTC= State of California AD Diagnostic and Treatment Centre Criteria; BKSCA-R= Brief Kingston Standardized Cognitive Assessment- Revised; BVRT= Benton´s Visual Retention Test; CAMCI= Chinese Abbreviated Mild Cognitive Impairment Test; CAMCOG= Cambridge Cognitive Examination; CASI= Cognitive Abilities Screening Instrument; CAST= Cognitive Assessment Screening Test; CCCE= Cross-cultural Cognitive Examination; CCSE= Cognitive Capacity Screening Examination; CERAD= Consortium to Establish a registry for Alzheimer´s Disease; CSF= Cerebrospinal fluid; CSI-D= Community Screening Instrument for Dementia; CT= Computed tomography; DaTSCAN= DaT, 123 I-FP-CT; DLB= Dementia with Lewy Bodies; DSM= Diagnostic and Statistical Manual of Mental Disorders; FDG-PET= PET using 2-Fluro-deoxy D-glucose; FTD= Fronto-Temporal Dementia; ICD-10= International Statistical Classification of Diseases and Related Health Problems 10th Revision; IQCODE= Informant Questionnaire on Cognitive Decline in the Elderly; IST= Isaacs Set Test; M@T= Memory Alteration Test; MCI= Mild cognitive Impairment; MDRS= Mattis Dementia Rating Scale; MFI= Mental Function Index; MIBG= 123 I-metaiodobenzylguanidine scintigraphy; MIS= Memory Impairment Screen; MMSE= Mini-Mental State Examination; MOCA= Montreal Cognitive Assessment; MRI= Magnetic Resonance Imaging; NCSE= Neurobehavioral Cognitive Screening Examination; NINCDS-ADRDA= National Institute of Neurological and Communicative Disorders and Stroke and the Alzheimer´s Disease and Related Disorders Association; NINDS-AIREN= National Institute of Neurological Disorders and Stroke and Association Internationales pour la Recherche et l´Ensignement en Neurosciences; NUCOG= Neuropsychiatry Unit Cognitive Assessment Tool; PIB-PET= 11 C-Pittsburgh Compound B- positron emission tomography; Ptau= Phosphorylated Tau; RBANS= Repeatable Battery for the Assessment of Neuropsychological Status; RUDAS= Rowland Universal Dementia Assessment Scale; SPECT= Single photon emission computed tomography; STMS= Short Test of Mental Status; TMT B= Trail making Test Part B; Ttau= Total Tau; VaD= Vascular Dementia.*

**4. DTA systematic reviews about dementia- AMSTAR items**

| **Review** | **AMSTAR 1** | **AMSTAR 2** | **AMSTAR 3** | **AMSTAR 4** | **AMSTAR 5** | **AMSTAR 6** | **AMSTAR 7** | **AMSTAR 8** | **AMSTAR 9** | **AMSTAR 10** | **AMSTAR 11** |
| --- | --- | --- | --- | --- | --- | --- | --- | --- | --- | --- | --- |
| Appels 2010 [[15](#_ENREF_15)] | No | No | No | No | No | Yes | Yes | Yes | NA | No | Yes |
| Beynon 2012 [[16](#_ENREF_16)] | Yes | Yes | Yes | Yes | No | Yes | Yes | Yes | Yes | NA | Yes |
| Bloudek 2011 [[17](#_ENREF_17)] | No | No | No | No | No | Yes | No | NA | Yes | No | Yes |
| Carnero 2003 [[18](#_ENREF_18)] | No | No | No | Yes | No | Yes | No | Yes | NA | No | No |
| Crawford 2012 [[19](#_ENREF_19)] | No | No | Yes | Yes | No | Yes | Yes | Yes | NA | No | Yes |
| Dougall 2004 [[20](#_ENREF_20)] | No | Yes | Yes | Yes | No | Yes | Yes | No | Yes | Yes | No |
| Ehreke 2010 [[21](#_ENREF_21)] | No | No | Yes | Yes | No | Yes | No | NA | NA | No | Yes |
| Ferrante 2004 [[22](#_ENREF_22)] | No | No | Yes | CA | No | Yes | No | Yes | CA | No | Yes |
| Lischka 2012 [[23](#_ENREF_23)] | No | Yes | Yes | No | No | Yes | Yes | No | NA | No | No |
| Lonie 2009 [[24](#_ENREF_24)] | No | No | Yes | No | No | Yes | No | NA | NA | No | Yes |
| Matchar 2001 [[25](#_ENREF_25)] | No | Yes | No | No | No | Yes | Yes | No | Yes | No | Yes |
| Mitchell 2009a [[27](#_ENREF_27)] | No | No | Yes | No | No | Yes | No | NA | CA | No | Yes |
| Mitchell 2009b [[26](#_ENREF_26)] | No | No | Yes | Yes | Yes | Yes | No | NA | No | No | No |
| Mitchell 2010a [[28](#_ENREF_28)] | No | No | Yes | Yes | No | Yes | No | NA | No | No | No |
| Mitchell 2010b [[29](#_ENREF_29)] | No | No | Yes | Yes | No | Yes | No | NA | No | No | No |
| Monge 2010 [[30](#_ENREF_30)] | No | No | No | Yes | No | Yes | No | NA | CA | No | No |
| Papathanasiou 2012 [[31](#_ENREF_31)] | No | No | No | No | Yes | Yes | No | NA | Yes | Yes | Yes |
| Patwardhan 2004 [[32](#_ENREF_32)] | No | Yes | Yes | No | No | Yes | Yes | Yes | No | No | No |
| Treglia 2012 [[33](#_ENREF_33)] | No | Yes | Yes | Yes | Yes | Yes | No | NA | CA | No | Yes |
| Van Harten 2011 [[34](#_ENREF_34)] | No | No | No | No | No | Yes | No | NA | CA | No | Yes |
| van Rossum 2010 [[35](#_ENREF_35)] | No | No | Yes | CA | No | Yes | No | NA | CA | No | Yes |
| Yeo 2013 [[38](#_ENREF_38)] | No | No | Yes | No | No | Yes | Yes | No | No | Yes | Yes |
| Yuan 2009 [[36](#_ENREF_36)] | No | Yes | No | No | No | Yes | Yes | Yes | CA | Yes | No |
| Zhang 2012 [[37](#_ENREF_37)] | No | Yes | Yes | No | No | Yes | Yes | Yes | Yes | Yes | No |

**5. DTA systematic reviews about dementia- PRISMA items**

| **Review** | **Item 1** | **Item 2** | **Item 3** | **Item 4** | **Item 5** | **Item 6** | **Item 7** | **Item 8** | **Item 9** | **Item 10** | **Item 11** | **Item 12** | **Item 13** | **Item 14** | **Item 15** | **Item 16** | **Item 17** | **Item 18** | **Item 19** | **Item 20** | **Item 21** | **Item 22** | **Item 23** | **Item 24** | **Item 25** | **Item 26** | **Item 27** |
| --- | --- | --- | --- | --- | --- | --- | --- | --- | --- | --- | --- | --- | --- | --- | --- | --- | --- | --- | --- | --- | --- | --- | --- | --- | --- | --- | --- |
| Appels 2010 [[15](#_ENREF_15)] | Yes | No | Yes | No | No | Yes | Yes | No | Yes | No | No | Yes | Yes | No | No | No | Yes | Yes | Yes | No | Yes | Yes | No | Yes | Yes | Yes | Yes |
| Beynon 2012 [[16](#_ENREF_16)] | Yes | Yes | Yes | Yes | Yes | Yes | Yes | Yes | Yes | Yes | Yes | Yes | Yes | Yes | Yes | Yes | Yes | Yes | Yes | Yes | Yes | No | Yes | Yes | Yes | Yes | Yes |
| Bloudek 2011 [[17](#_ENREF_17)] | Yes | Yes | Yes | Yes | No | No | Yes | Yes | No | No | No | No | Yes | Yes | No | Yes | Yes | No | No | Yes | Yes | No | Yes | No | No | No | Yes |
| Carnero 2003 [[18](#_ENREF_18)] | Yes | Yes | Yes | No | No | Yes | No | Yes | No | No | No | No | No | No | No | No | Yes | Yes | No | No | No | No | No | Yes | No | No | No |
| Crawford 2012 [[19](#_ENREF_19)] | Yes | Yes | Yes | Yes | No | Yes | Yes | No | Yes | No | No | Yes | Yes | No | No | No | Yes | Yes | Yes | No | No | No | No | Yes | Yes | Yes | No |
| Dougall 2004 [[20](#_ENREF_20)] | Yes | Yes | Yes | Yes | No | No | No | No | No | Yes | No | Yes | Yes | Yes | Yes | Yes | Yes | Yes | Yes | Yes | Yes | No | No | No | No | No | Yes |
| Ehreke 2010 [[21](#_ENREF_21)] | Yes | Yes | Yes | No | No | No | No | No | No | No | No | No | No | No | No | No | Yes | Yes | No | No | No | No | No | No | No | No | Yes |
| Ferrante 2004 [[22](#_ENREF_22)] | No | Yes | Yes | Yes | No | Yes | No | Yes | No | No | No | No | Yes | No | No | No | Yes | No | No | Yes | Yes | No | No | Yes | No | No | Yes |
| Lischka 2012 [[23](#_ENREF_23)] | Yes | Yes | Yes | Yes | No | Yes | Yes | No | Yes | Yes | Yes | Yes | No | No | No | No | Yes | Yes | Yes | No | No | No | No | No | No | Yes | No |
| Lonie 2009 [[24](#_ENREF_24)] | Yes | Yes | Yes | No | No | No | Yes | No | No | No | No | No | No | No | No | No | Yes | Yes | No | No | No | No | No | No | No | Yes | Yes |
| Matchar 2001 [[25](#_ENREF_25)] | No | Yes | Yes | Yes | No | Yes | Yes | Yes | Yes | Yes | No | Yes | Yes | Yes | No | No | Yes | Yes | Yes | Yes | Yes | No | No | No | No | No | Yes |
| Mitchell 2009a [[27](#_ENREF_27)] | Yes | No | Yes | Yes | Yes | Yes | Yes | No | No | No | No | No | Yes | No | No | No | Yes | Yes | No | Yes | Yes | No | No | No | No | Yes | Yes |
| Mitchell 2009b [[26](#_ENREF_26)] | Yes | No | Yes | Yes | No | No | Yes | No | No | No | No | No | Yes | Yes | No | No | Yes | Yes | No | Yes | Yes | No | No | No | Yes | Yes | No |
| Mitchell 2010a [[28](#_ENREF_28)] | Yes | Yes | Yes | Yes | No | Yes | Yes | Yes | No | No | No | No | Yes | Yes | Yes | No | Yes | Yes | No | No | Yes | No | No | No | No | No | No |
| Mitchell 2010b [[29](#_ENREF_29)] | Yes | Yes | Yes | Yes | No | Yes | Yes | Yes | No | No | No | No | Yes | Yes | Yes | No | Yes | Yes | No | Yes | Yes | No | No | No | No | No | No |
| Monge 2010 [[30](#_ENREF_30)] | Yes | Yes | Yes | Yes | No | Yes | Yes | No | No | No | No | No | Yes | Yes | No | No | Yes | No | No | Yes | Yes | No | No | No | Yes | Yes | No |
| Papathanasiou 2012 [[31](#_ENREF_31)] | Yes | Yes | Yes | Yes | No | Yes | Yes | No | No | No | No | No | Yes | Yes | Yes | No | Yes | Yes | No | Yes | Yes | Yes | No | Yes | Yes | Yes | No |
| Patwardhan 2004 [[32](#_ENREF_32)] | Yes | Yes | Yes | Yes | No | Yes | Yes | No | Yes | Yes | No | Yes | Yes | Yes | No | No | Yes | Yes | Yes | Yes | Yes | No | No | Yes | Yes | Yes | Yes |
| Treglia 2012 [[33](#_ENREF_33)] | Yes | Yes | Yes | Yes | No | Yes | Yes | No | Yes | Yes | Yes | No | Yes | Yes | No | No | Yes | Yes | No | Yes | Yes | No | No | Yes | Yes | Yes | No |
| Van Harten 2011 [[34](#_ENREF_34)] | Yes | Yes | Yes | Yes | No | Yes | Yes | No | No | No | No | No | Yes | Yes | No | No | No | Yes | No | Yes | Yes | No | No | Yes | Yes | Yes | Yes |
| van Rossum 2010 [[35](#_ENREF_35)] | No | No | Yes | No | No | No | No | No | No | No | No | No | Yes | Yes | No | No | No | Yes | No | No | Yes | No | No | Yes | Yes | Yes | Yes |
| Yeo 2013 [[38](#_ENREF_38)] | Yes | Yes | Yes | No | No | Yes | Yes | No | Yes | No | Yes | Yes | Yes | Yes | Yes | Yes | Yes | Yes | Yes | Yes | Yes | Yes | No | Yes | Yes | Yes | No |
| Yuan 2009 [[36](#_ENREF_36)] | Yes | Yes | Yes | No | No | Yes | No | No | Yes | Yes | Yes | Yes | Yes | Yes | Yes | Yes | Yes | Yes | No | No | Yes | Yes | Yes | Yes | Yes | Yes | Yes |
| Zhang 2012 [[37](#_ENREF_37)] | No | Yes | Yes | Yes | No | Yes | Yes | No | Yes | Yes | Yes | Yes | Yes | Yes | Yes | Yes | Yes | Yes | Yes | Yes | Yes | Yes | Yes | Yes | Yes | Yes | No |

**6. List of excluded studies**

1. Agarwal R, Tripathi CB: **Diagnostic Utility of CSF Tau and Abeta(42) in Dementia: A Meta-Analysis**. *International journal of Alzheimer's disease* 2011, **2011**:503293.

2. Boustani M, Peterson B, Harris R, Lux LJ, Krasnov C, Sutton SF, Hanson L, Lohr KN: **Screening for Dementia**. Rockville MD; 2003.

3. Budd D, Burns L, Berman R, L'Italien G, Nalysnyk L, Fahrbach K: **Examining CSF biomarkers and their ability to predict dementia, a systematic review of the literature**. *Alzheimer's and Dementia* 2011, **7**(4):S314.

4. Budd D, Nalysnyk L, Fahrbach K, Bhagwagar Z, Berman R, Burns L, L'Italien G: **VMRI and hippocampalvolumein patients with suspected predementia Alzheimer's disease**. *Alzheimer's and Dementia* 2012, **8**(4):P411.

5. Carnero-Pardo C, Martinez BE, Montoro-Rios MT: **Systematic review and meta-analyses on the diagnostic accuracy of the Phototest for dementia screening**. *European Journal of Neurology* 2009, **16**(S3):344.

6. Clerx L, Visser PJ, Verhey F, Aalten P: **New MRI markers for Alzheimer's disease: a meta-analysis of diffusion tensor imaging and a comparison with medial temporal lobe measurements**. *Journal of Alzheimer's disease : JAD* 2012, **29**(2):405-429.

7. Davison CM, O'Brien JT: **A comparison of FDG-PET and blood flow SPECT in the diagnosis of neurodegenerative dementias: a systematic review**. *Int J Geriatr Psychiatry* 2013.

8. Daly MP: **Diagnosis and management of Alzheimer disease**. *The Journal of the American Board of Family Practice / American Board of Family Practice* 1999, **12**(5):375-385.

9. Debette S, Markus HS: **The clinical importance of white matter hyperintensities on brain magnetic resonance imaging: systematic review and meta-analysis**. *BMJ (Clinical research ed)* 2010, **341**:c3666.

10. Diniz BS, Pinto Junior JA, Forlenza OV: **Do CSF total tau, phosphorylated tau, and beta-amyloid 42 help to predict progression of mild cognitive impairment to Alzheimer's disease? A systematic review and meta-analysis of the literature**. *The world journal of biological psychiatry : the official journal of the World Federation of Societies of Biological Psychiatry* 2008, **9**(3):172-182.

11. Dukart J, Mueller K, Barthel H, Villringer A, Sabri O, Schroeter ML: **Meta-analysis based SVM classification enables accurate detection of Alzheimer's disease across different clinical centers using FDG-PET and MRI**. *Psychiatry research* 2012.

12. Durand-Martel P, Tremblay D, Brodeur C, Paquet N: **Autopsy as gold standard in FDG-PET studies in dementia**. *Canadian Journal of Neurological Sciences* 2010, **37**(3):336-342.

13. Elias-Sonnenschein LS, Viechtbauer W, Ramakers IH, Verhey FR, Visser PJ: **Predictive value of APOE-epsilon4 allele for progression from MCI to AD-type dementia: a meta-analysis**. *Journal of neurology, neurosurgery, and psychiatry* 2011, **82**(10):1149-1156.

14. Espejo-Martinez B, Carnero-Pardo C, Montoro-Rios MT: **Systematic review and meta-analysis of the accuracy of the eurotest for the detection of dementia**. *Alzheimer's and Dementia* 2009, **5**(4):447.

15. Ferreira LK, Diniz BS, Forlenza OV, Busatto GF, Zanetti MV: **Neurostructural predictors of Alzheimer's disease: a meta-analysis of VBM studies**. *Neurobiology of aging* 2011, **32**(10):1733-1741.

16. Formichi P, Battisti C, Radi E, Federico A: **Cerebrospinal fluid tau, A(beta), and phosphorylated tau protein for the diagnosis of Alzheimer's disease**. *Journal of Cellular Physiology* 2006, **208**(1):39-46.

17. Gifford DR, Holloway RG, Vickrey BG: **Systematic review of clinical prediction rules for neuroimaging in the evaluation of dementia**. *Archives of internal medicine* 2000, **160**(18):2855-2862.

18. Gill SS, Rochon PA, Guttman M, Laupacis A: **The value of positron emission tomography in the clinical evaluation of dementia**. *Journal of the American Geriatrics Society* 2003, **51**(2):258-264.

19. Killen AR, Oster G, Colditz GA: **An assessment of the role of 123I-N-isopropyl-p-iodoamphetamine with single-photon emission computed tomography in the diagnosis of stroke and Alzheimer's disease**. *Nuclear medicine communications* 1989, **10**(4):271-284.

20. King AE, Mintz J, Royall DR: **Meta-analysis of 123I-MIBG cardiac scintigraphy for the diagnosis of Lewy body-related disorders**. *Movement disorders : official journal of the Movement Disorder Society* 2011, **26**(7):1218-1224.

21. Koyama A, Okereke O, Yang T, Selkoe D, Grodstein F: **Plasma amyloid-beta as a predictor of dementia and cognitive decline: A systematic reviewand meta-analysis**. *Alzheimer's and Dementia* 2011, **7**(4):S318.

22. Lim X, Yeo JM, Green A, Pal S: **The diagnostic utility of cerebrospinal fluid alpha-synuclein analysis in dementia with Lewy bodies - a systematic review and meta-analysis**. *Parkinsonism & related disorders* 2013, **19**(10):851-858.

23. Liu B, Tang Y, Shen Y, Cen L, Han M: **Cerebrospinal Fluid tau Protein in Differential Diagnosis of Alzheimer's Disease and Vascular Dementia in Chinese population: A Meta-Analysis**. *American journal of Alzheimer's disease and other dementias* 2013.

24. Mathias JL, Burke J: **Cognitive functioning in Alzheimer's and vascular dementia: a meta-analysis**. *Neuropsychology* 2009, **23**(4):411-423.

25. McShane R, Noel-Storr A, Ritchie C, Flicker L: **The qualityand extent of evidence for biomarkers: A cochrane systematic review**. *Alzheimer's and Dementia* 2011, **7**(4):S100-S101.

26. Mitchell AJ: **The clinical significance of subjective memory complaints in the diagnosis of mild cognitive impairment and dementia: a meta-analysis**. *Int J Geriatr Psychiatry* 2008, **23**(11):1191-1202.

27. Monge-Argiles A: **Meta-analysis of CSF biomarkers in mild cognitive impairment patients: Its predictive value for Alzheimer's disease diagnosis**. *Alzheimer's and Dementia* 2009, **5**(4):345.

28. Moroney JT, Bagiella E, Desmond DW, Hachinski VC, Molsa PK, Gustafson L, Brun A, Fischer P, Erkinjuntti T, Rosen W *et al*: **Meta-analysis of the Hachinski Ischemic Score in pathologically verified dementias**. *Neurology* 1997, **49**(4):1096-1105.

29. Mosconi L, De Santi S, Rusinek H, Convit A, De Leon MJ: **Magnetic resonance and PET studies in the early diagnosis of Alzheimer's disease**. *Expert Rev Neurother* 2004, **4**(5):831-849.

30. Noel-Storr AH, Flicker L, Ritchie CW, Nguyen GH, Gupta T, Wood P, Walton J, Desai M, Solomon DF, Molena E *et al*: **Systematic review of the body of evidence for the use of biomarkers in the diagnosis of dementia**. *Alzheimer's & dementia : the journal of the Alzheimer's Association* 2013, **9**(3):e96-e105.

31. Pan PL, Shi HC, Zhong JG, Xiao PR, Shen Y, Wu LJ, Song YY, He GX, Li HL: **Gray matter atrophy in Parkinson's disease with dementia: evidence from meta-analysis of voxel-based morphometry studies**. *Neurological sciences : official journal of the Italian Neurological Society and of the Italian Society of Clinical Neurophysiology* 2012.

32. Pan PL, Song W, Yang J, Huang R, Chen K, Gong QY, Zhong JG, Shi HC, Shang HF: **Gray matter atrophy in behavioral variant frontotemporal dementia: a meta-analysis of voxel-based morphometry studies**. *Dementia and geriatric cognitive disorders* 2012, **33**(2-3):141-148.

33. Peters R, Pinto EM: **Predictive value of the clock drawing test: A review of the literature**. *Dementia and geriatric cognitive disorders* 2008, **26**(4):351-355.

34. Petzold A, Keir G, Warren J, Fox N, Rossor MN: **A systematic review and meta-analysis of CSF neurofilament protein levels as biomarkers in dementia**. *Neuro-degenerative diseases* 2007, **4**(2-3):185-194.

35. Pinto E, Peters R: **Literature review of the Clock Drawing Test as a tool for cognitive screening**. *Dementia and geriatric cognitive disorders* 2009, **27**(3):201-213.

36. Quigley H, Colloby SJ, O'Brien JT: **PET imaging of brain amyloid in dementia: A review**. *Int J Geriatr Psychiatry* 2011, **26**(10):991-999.

37. Ritchie C, Flicker L, Smailagic N, Noel AS, McShane R: **Plasma and CSF A(beta) for the diagnosis of Alzheimer's disease dementia and other dementias in mild cognitive impairment: A cochrane systematic review**. *Alzheimer's and Dementia* 2012, **8**(4):P266.

38. Schmand B, Huizenga HM, van Gool WA: **Meta-analysis of CSF and MRI biomarkers for detecting preclinical Alzheimer's disease**. *Psychological medicine* 2010, **40**(1):135-145.

39. Schmitt FA, Wichems CH: **A systematic review of assessment and treatment of moderate to severe Alzheimer's disease**. *Primary care companion to the Journal of clinical psychiatry* 2006, **8**(3):158-159.

40. Schroeter ML, Neumann J: **Combined Imaging Markers Dissociate Alzheimer's Disease and Frontotemporal Lobar Degeneration - An ALE Meta-Analysis**. *Frontiers in aging neuroscience* 2011, **3**:10.

41. Schwindt GC, Black SE: **Functional imaging studies of episodic memory in Alzheimer's disease: a quantitative meta-analysis**. *NeuroImage* 2009, **45**(1):181-190.

42. Sikkes SA, de Lange-de Klerk ES, Pijnenburg YA, Scheltens P, Uitdehaag BM: **A systematic review of Instrumental Activities of Daily Living scales in dementia: room for improvement**. *Journal of neurology, neurosurgery, and psychiatry* 2009, **80**(1):7-12.

43. Song F, Poljak A, Valenzuela M, Mayeux R, Smythe GA, Sachdev PS: **Meta-analysis of plasma amyloid-(beta) levels in alzheimer's disease**. *Journal of Alzheimer's Disease* 2011, **26**(2):365-375.

44. Sullivan SD, Bloudek L, Spackman DE, Blankenburg M: **A meta-analysis of biomarkers and diagnostic imaging in alzheimer's disease**. *Value in Health* 2011, **14**(3):A79.

45. Treglia G, Cason E, Stefanelli A, Cocciolillo F, Di Giuda D, Fagioli G, Giordano A: **MIBG scintigraphy in differential diagnosis of Parkinsonism: a meta-analysis**. *Clinical autonomic research : official journal of the Clinical Autonomic Research Society* 2012, **22**(1):43-55.

46. Wahlund LO, Almkvist O, Blennow K, Engedahl K, Johansson A, Waldemar G, Wolf H: **Evidence-based evaluation of magnetic resonance imaging as a diagnostic tool in dementia workup**. *Topics in magnetic resonance imaging : TMRI* 2005, **16**(6):427-437.

47. Watson R, Blamire AM, O'Brien JT: **Magnetic resonance imaging in lewy body dementias**. *Dementia and geriatric cognitive disorders* 2009, **28**(6):493-506.

48. Wild K, Howieson D, Webbe F, Seelye A, Kaye J: **Status of computerized cognitive testing in aging: a systematic review**. *Alzheimer's & dementia : the journal of the Alzheimer's Association* 2008, **4**(6):428-437.

49. Yang J, Pan P, Song W, Huang R, Li J, Chen K, Gong Q, Zhong J, Shi H, Shang H: **Voxelwise meta-analysis of gray matter anomalies in Alzheimer's disease and mild cognitive impairment using anatomic likelihood estimation**. *Journal of the neurological sciences* 2012, **316**(1-2):21-29.

50. Zakzanis KK: **Quantitative evidence for neuroanatomic and neuropsychological markers in dementia of the Alzheimer's type**. *Journal of clinical and experimental neuropsychology* 1998, **20**(2):259-269.

51. Zakzanis KK, Graham SJ, Campbell Z: **A meta-analysis of structural and functional brain imaging in dementia of the Alzheimer's type: a neuroimaging profile**. *Neuropsychology review* 2003, **13**(1):1-18.

52. Schroeter ML, Stein T, Maslowski N, Neumann J: **Neural correlates of Alzheimer's disease and mild cognitive impairment: a systematic and quantitative meta-analysis involving 1351 patients**. *NeuroImage* 2009, **47**(4):1196-1206.
